# Inhibitory Effect of *Lactiplantibacillus plantarun* HFY11 on Compound Diphenoxylate-Induced Constipation in Mice

**DOI:** 10.3390/biom15030358

**Published:** 2025-03-01

**Authors:** Fang Tan, Chang-Suk Kong

**Affiliations:** 1 Department of Bioscience, Silla University, Busan 46958, Republic of Korea; g-202311663@sillain.ac.kr; 2 Department of Food Science and Nutrition, Silla University, Busan 46958, Republic of Korea; 3 Marine Biotechnology Center for Pharmaceuticals and Foods, Silla University, Busan 46958, Republic of Korea

**Keywords:** probiotics, intestinal tract, hormone, expression, cytokine

## Abstract

*Lactiplantibacillus plantarun* HFY11 (LP-HFY11) is a newly discovered microbial strain. This study was the first to investigate the preventive effect of LP-HFY11 on compound diphenoxylate induced constipation in mice by measuring intestinal contents, serum, and small intestinal tissue indexes. In mice suffering from constipation, LP-HFY11 could prevent the reduction in fecal weight, particle count, and water content. The constipated mice that ingested a high LP-HFY11 dose (LP-HFY11H) expelled the first black stool faster than the model group and the drug lactulose-treated group, but they were slower than the normal group. Furthermore, the small intestine in the LP-HFY11H group had a greater propulsion rate of activated charcoal than that in the model and lactulose groups, but the propulsion rate was still lower than that in the normal group. According to hematoxylin–eosin (H&E) staining, LP-HFY11H was more effective than lactulose at reducing intestinal villi breaking and constipation-induced harm to the small intestine. Simultaneously, compared with the model group, the LP-HFY11H group had markedly increased serum levels of motilin (MTL), endothelin-1 (ET-1), vasoactive intestinal peptide (VIP), and acetylcholinesterase (AchE). Transient receptor potential vanilloid 1 (TRPV1) expression was only higher than in the normal group, but the mRNA expression of c-Kit, stem cell factor (SCF), and glial cell line-derived neurotrophic factor (GDNF) was all higher in the small intestine in the LP-HFY11H group than in the model and lactulose groups, according to the results of quantitative polymerase chain reaction (qPCR) experiments. Analysis of microbial mRNA in the small intestinal contents of the constipated mice further validated the capacity of LP-HFY11 to decrease the abundance of *Firmicutes* and increase the abundance of *Bacteroidetes*, *Bifidobacteria*, and *Lactobacillus*. This revealed that LP-HFY11, which produced better results than the drug lactulose, can control the gut microbiota of constipated mice and successfully cure constipation. LP-HFY11 has the potential to be used as a probiotic in the treatment of constipation. It has good application prospects in the food industry and biopharma.

## 1. Introduction

Constipation is a now common physiological condition in which people have fewer bowel movements and trouble defecating because their stool contains less water [1]. Owing to work conditions and other factors, approximately 70% of the population belongs to a sub-healthy state, characterized by gastrointestinal discomfort, and a considerable amount of this demographic experience constipation. According to the World Gastroenterology Organisation, the prevalence of chronic constipation is as high as 20%, rising to 50% in the elderly, and reaching 67% in people over 65 years of age. Constipation, as a common chronic gastrointestinal disease, has a high prevalence and long course of disease. It is often combined with sleep disorders, anxiety, depression, and physical and mental symptoms. The interaction between physical symptoms and mental symptoms reduces work and study efficiency, leads to interpersonal communication disorders, social function decline, and damage the quality of life of patients. Due to the high consultation rate of patients with constipation, medical resources are consumed, medical expenditure is increased, and the economic burden is heavy. Constipation has an impact on the quality of life and economic burden of patients. Clinicians and the general population should pay attention to the prevention, diagnosis and treatment of constipation. Chronic constipation may cause excessive dilation of the colon and affect its normal peristalsis. When the intestinal transit time is prolonged during constipation, harmful bacteria may overgrow in the gut, whereas the proportion of beneficial bacteria may decrease, which then affects intestinal health [2]. The intestine is a crucial organ for detoxification; constipation may lead to toxin accumulation, thereby influencing the function of other organs. Chronic constipation is linked to a higher risk of medical conditions such as inflammatory bowel disease and colorectal cancer [3]. Consumption of functional foods can improve physical conditions and functions, thereby facilitating individuals in a sub-healthy state to return to normal. Thus, this method has been currently promoted as crucial for augmenting intestinal health [4]. The commonly used chemical drugs for the treatment of constipation come with certain side effects, including nausea, abdominal pain, headache, insomnia, diarrhea, and even cardiac discomfort. Long-term use may also lead to the body becoming dependent on the drug. Some natural products with minimal side effects may also cause intestinal dependence and potential liver damage after long-term use. Probiotics can regulate the balance of intestinal microorganisms and contribute to intestinal peristalsis, thus relieving constipation. Probiotics play an intervention role in constipation by promoting intestinal health, which not only has no side effects, but also promotes the health of the body by inhibiting constipation and related diseases. It is of great significance to screen natural probiotics. In this study, high-quality probiotics were extracted from naturally fermented food in high altitude areas, aiming to find non-toxic and harmless probiotics that can regulate intestinal health, which is easy to use and does not cause economic burden, and provide a new solution to improve constipation.

Probiotics can offer various benefits for the host within the body. They improve and maintain the intestinal microenvironment through multiple mechanisms, thus exerting a positive intervention effect on constipation symptoms [5]. By competitively excluding pathogens, producing antimicrobial substances, and regulating pH in the gut, probiotics help increase the population of beneficial bacteria and reduce the growth of harmful bacteria [6]. Additionally, probiotics can increase peristalsis and promote smooth muscle contraction in the intestine by generating short-chain fatty acids, particularly butyrate, which then facilitate the flow of intestinal contents and relieve constipation [7]. Probiotics benefit the general dynamics of the gut. They can control gastrointestinal motility, which involves stimulating the enteric nervous system, and enhancing the sensory and motor responses of the gut. The intestine is a source of innumerable hormones with a regulatory role in gastrointestinal movement and responses. Probiotics may impact the secretion of these hormones, thereby affecting defecation [8].

Lactulose is a drug used to treat chronic functional constipation. It helps to keep the stool open by increasing intestinal moisture and stimulating intestinal motility. It is not absorbed by the body and has high safety [9]. In an experiment for determining the effectiveness of bioactive compounds in relieving constipation, the pharmacological activity of diphenoxylate suspension is used to induce constipation in mice, thus establishing an animal constipation model [10]. The present study assessed the ability of *Lactiplantibacillus plantarun* HFY11 (LP-HFY11) to prevent loperamide-induced constipation. Through animal experiments, this study verified the effect of the probiotic LP-HFY11 on intestinal dynamics and evaluated its potential in regulating intestinal flora composition, boosting intestinal peristalsis capacity, and maintaining the function of the intestinal wall barrier. In particular, the study sought to promote enhancements in constipation regulation from the intestinal microbiology perspective. Based on the study findings, we intend to offer a new therapeutic avenue for individuals with constipation to augment their treatment experience and outcomes.

## 2. Materials and Methods

### 2.1. Isolation and Culture of Microorganisms

Traditional naturally fermented yak yogurt was collected from homes of herdsmen in the Hongyuan County area of the Aba Tibetan and Qiang Autonomous Prefecture, Sichuan Province, China. After thoroughly stirring the yogurt with a sterile spoon, 50 mL of yogurt was sucked into a sterilized centrifuge tube with a sterile syringe, and then placed in a low-temperature food sampling box to bring back to the laboratory and stored in an ultra-low temperature refrigerator at −80 °C for later use. The yogurt samples were diluted and plated on DeMan, Rogosa, and Sharpe-sodium thioglycolate (MRS-THIO) medium. After forming colonies, different colonies on the plate were picked and separated, and the above procedures were repeated several times. Until pure single colonies of consistent morphology are obtained. It was identified and named LP-HFY11, and was found to have a survival rate of more than 80% in pH 3.0 artificial gastric juice and a growth efficiency of more than 50% in 0.3% bile salt [11], it can be considered that it has probiotic potential. The isolate was preserved at the China General Microbiological Culture Collection Center (Preservation number: CGMCC No. 16644, Beijing, China) and was then freeze-dried to prepare lyophilized bacterial powder for use. MRS-THIO medium was used for LP-HFY11 expansion culture at 37 °C for 72 h. The fermented bacteria were centrifuged by ultracentrifuge at 4500 r/min for 20 min, and the supernatant was removed for sediment collection. The sediment collected in the first step was mixed with an oscillator to make bacterial suspension. Then, it was placed in a freezer at 5 °C for 45 min, then frozen at −30 °C for 60 min, and then frozen at −80 °C for 60 min. Then, it was freeze-dried on the lyophilizer for 15 h, and then sealed and stored in a freezer at 4 °C. Vacuum freeze-drying conditions were set at the temperature of −60 °C, pressure of 0.006 Pa, and time of 14 h to obtain the lyophilized powder of lactic acid bacteria for the test. The diet was supplemented with lyophilized bacterial powder in proportion for subsequent experiments.

### 2.2. Experimental Microorganisms

*Lactiplantibacillus plantarun* YS-3 and *Lactiplantibacillus plantarun* CQPC10 are two strains that have been proved to have good anti-constipation effects through animal experiments and are stored in China Center for Type Culture Collection (Wuhan, Hubei, China, preservation number: CCTCC No. M 2016749) and China General Microbiological Culture Collection Center (preservation number: CGMCC No. 14959), respectively [12,13]. In this study, these two strains were selected as comparative probiotic strains for the preliminary effect comparison of LP-HFY11.

### 2.3. Animal Experiment

**Figure 1:**
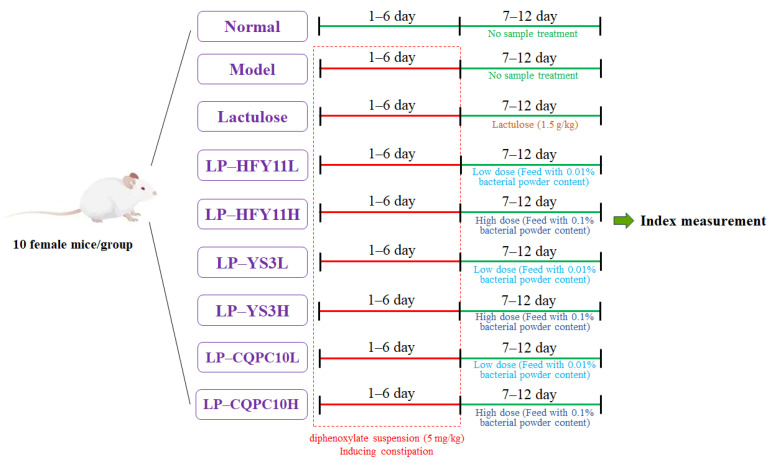
Procedure of animal experiment in this study.

The incidence of constipation in females is 2–3 times higher than that in males. Therefore, female mice were used in this study. Ninety specific-pathogen-free female ICR mice (age: 7 weeks, weight: 25–30 g) [Hunan Slake Jingda Experimental Animal Co., Ltd., Changsha, Hunan, China, animal permit number: SCXK (Xiang) 2019-0004] were evenly and randomized into nine groups, with 10 mice per group: a high-concentration treatment group with LP-HFY11 (LP-HFY11H), a low-concentration treatment group with LP-HFY11 (LP-HFY11L), a lactulose group (drug positive control group), a high-concentration treatment group with LP-YS3 (LP-YS3H), a low-concentration treatment group with LP-YS3 (LP-YS3L), a lactulose group (drug positive control group), a high-concentration treatment group with LP-CQPC10 (LP-CQPCH), a low-concentration treatment group with LP-YS3 (LP-YS3L), a lactulose group (drug positive control group), a model group, and a normal group (Figure 1). The other group of mice, except for mice from the normal group, were orally administered diphenoxylate suspension (5 mg/kg) once daily for 6 consecutive days. Lactulose (1.5 g/kg) was orally administered to the mice in the lactulose treatment group once daily for the complete duration of the trial, starting from 24 h after the induction of constipation. The LP-HFY11L, LP-YS3L or LP-CQPC10L groups had free access to food containing 0.01% (*w*/*w*, 10^7^ CFU of bacterial powder/g feed) of the LP-HFY11, LP-YS3 or LP-CQPC10 lyophilized bacterial powder; the LP-HFY11H, LP-YS3H or LP-CQPC10H groups had free access to food containing 0.1% (*w*/*w*, 10^8^ CFU of bacterial powder/g feed) of the LP-HFY11, LP-YS3 or LP-CQPC10 lyophilized bacterial powder, respectively, and water ad libitum. For 6 days, this food was sustained. The mice were then starved for a whole day. Then, 10 mice per group were divided in half; five mice were observed to determine how long they took to expel the first black feces, and the other five mice were killed through cervical dislocation 30 min after they were administered a 10% activated charcoal ice water solution at 0.1 mL/10 g to determine how rapidly the charcoal was propelled through the small intestine. Blood and tissue samples were collected and examined to observe the effect of LP-HFY11L intervention on experimental constipation in mice. The propulsion rate (%) was equal to (distance traveled by the activated charcoal in the small intestine/total length of the small intestine) × 100 [14]. During the experiment, the weight, dietary intake, water intake, fecal weight, and fecal humidity of all mice were measured at 9:00 a.m. every day. The animal experiment was approved by the Animal Experiment Ethics Committee of Collaborative Innovation Center for Child Nutrition and Health Development, Chongqing University of Education (Chongqing, China) on 8 May 2024, the approval code is 202405011B.

### 2.4. Serum Biomarker Analysis

MTL is a gastrointestinal hormone, ET-1 is an effective autocrine and paracrine peptide, VIP is both a gastrointestinal hormone and a neuropeptide, and AchE is a key enzyme in biological nerve conduction. All the above indicators can reflect the changes in gastrointestinal function very precisely. The mouse blood was centrifuged (4500 rpm) for 10 min. The supernatant was collected to obtain serum. The levels of MTL, ET-1, VIP, and AchE in the mouse serum were measured using the respective kits (Thermo Fisher Scientific, Waltham, MA, USA).

### 2.5. Pathological Observation

After the mice were dissected, the colons were fixed in 10% formalin solution for 24 h. The colon tissues were dehydrated in 95% ethanol and clarified through immersion in xylene to replace ethanol within the tissue blocks, thereby rendering the tissues transparent. Next, the transparent blocks were embedded in molten paraffin wax for infiltration and then segmented using a microtome. The intestinal slices were stained with hematoxylin–eosin (H&E), prepared into pathological slides, and were observed under a microscope (BX53, Olympus, Tokyo, Japan) at 20× and 40× magnification [15].

### 2.6. Quantitative Polymerase Chain Reaction Analysis of Small Intestine Tissue Gene Expression

First, 100 mg of the middle segments of the small intestine tissues were extracted. The tissues were rinsed with physiological saline and further diluted with a new physiological saline at a 1:9 ratio. Then, 1.0 mL of the RNAzol reagent was used to extract RNA from the homogenized tissues. The concentration of the extracted RNA was adjusted to 1 μg/μL, and cDNA was generated from the extracted RNA through reverse transcription. Subsequently, 1 μL of cDNA, 10 μL of SYBR Green PCR Master Mix, 7 μL of sterile distilled water, and 1 μL of each of 10 µmol/L forward and reverse primers (Thermo Fisher Scientific) were added to the reaction mixture. The reaction mixture was then amplified using SteponePlus from Thermo Fisher Scientific under the following conditions: 30 s of initial denaturation at 95 °C, 40 cycles of denaturation at 95 °C for 15 s, annealing at 55 °C for 30 s, and extension at 72 °C for 35 s. GAPDH was used as the internal reference gene (Table 1), and the 2^−ΔΔCt^ method was employed to establish the relative expression level of each gene [16].

### 2.7. Determination of Microbial mRNA Expression in Mouse Intestinal Contents

First, 1.0 g of the mouse’s digestive contents was weighed. To evaluate the microbiota composition of the intestinal contents, microbial mRNA expression in these contents was assessed using the same techniques that are employed for measuring mRNA in the small intestine tissue.

### 2.8. Statistical Analysis

Each experiment was repeated thrice. The experimental results were analyzed using SAS 9.4 statistical software to assess whether the collected data had any significant differences at *p* < 0.05. In this analysis, one-way analysis of variance (ANOVA) was employed.

## 3. Results

### 3.1. Bowel Movements in Mice

The mice in the normal group had the largest fecal weight, particle number, and water content, whereas the mice in the constipation control group had the lowest, as seen in Table 2. Lactulose and LP-HFY11 were able to significantly mitigate the constipation-induced reduction in fecal weight, particle number, and water content (*p* < 0.05). Constipated mice’s fecal weight, particle number, and water content were the closest to those of the normal group thanks to LP-HFY11H. Lactulose is a commonly used constipation drug. The clinical dosage was calculated for experimental mice in this experiment, and the high dose of LP-HFY11 (LP-HFY11H) was the recommended intake of probiotics. It can be seen that the recommended intake of probiotics is stronger than the dosage of lactulose. At the same time, the effect of LP-HFY11 at both high and low doses is better than that of LP-YS3, which has been proved to have constipation inhibition effect, and the effect is close to that of LP-CQPC10. Therefore, it can be preliminarily concluded that LP-HFY11 has the same research value as the probiotics LP-YS3 and LP-CQPC10, which have been proved to have a good constipation effect and can be followed up.

### 3.2. Time to First Black Fecal Pellet Expulsion in Mice

**Figure 2:**
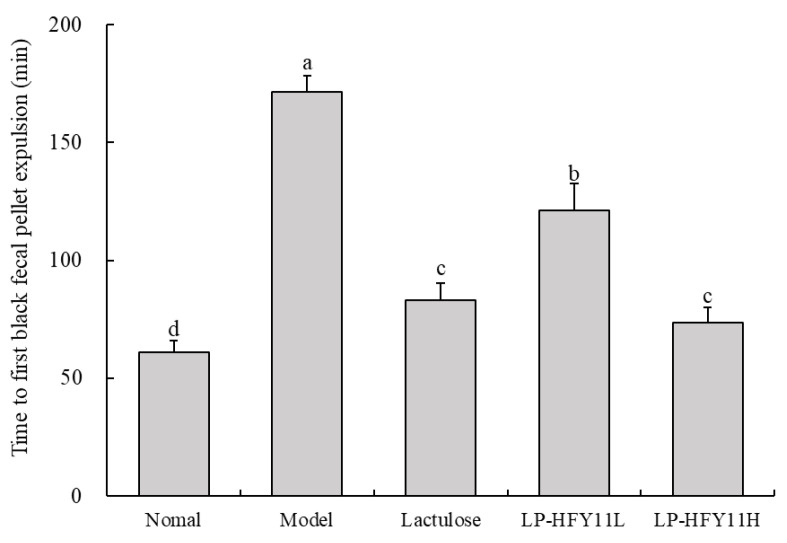
The time to first black fecal pellet expulsion in mice (*n* = 5). ^a–d^ The data mean values between the groups show statistically significant differences (*p* < 0.05) indicated by the letters.

After the mice were fasted for 24 h, they were gavaged with activated charcoal water. The time the mice took to expel the first black fecal pellet was noted. The normal group took the shortest time to expel the fecal pellet, which was 62 min (Figure 2). By contrast, the model group took the longest time to expulsion (172 min). Lactulose, a drug effective for constipation treatment in clinics, was used here as the treatment control for gavaging the mice. The time taken by the lactulose group to expel their fecal pellet decreased significantly compared with the model group (82 min). Similarly, LP-HFY11 could also reduce the expulsion time in the mice. The expulsion time for the LP-HFY11H group was 75 min, which was shorter than that for the lactulose (general drug dosage) group.

### 3.3. Activated Charcoal Propulsion Rate

Table 3 shows that the normal group had the largest propulsion distance and rate of activated charcoal in their small intestines, markedly outperforming the other groups (*p* < 0.05). The model group exhibited the lowest propulsion distance and rate because the mice in this group were constipated. The lactulose (general drug dosage) group exhibited a significant increase in the propulsion distance and rate compared with the model group, and the constipation of mice in this group was relieved because of the drug’s therapeutic action. However, these measures were still lower than those of the normal and LP-HFY11H groups.

### 3.4. Pathological Observation of the Mouse Colon

**Figure 3:**
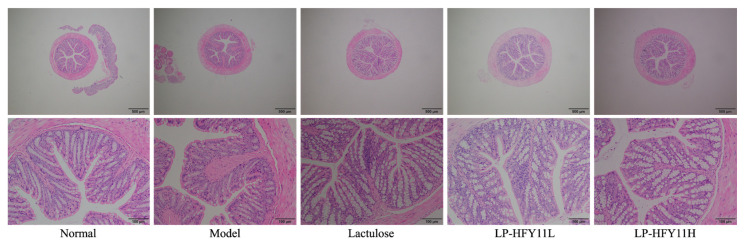
Pathological observation of mouse colon tissue.

The findings of the pathological observation demonstrated that the colon tissue of the mice in the normal group had intact mucosal epithelial cells, with glands dispersed neatly, normal crypts, and no ulcers (Figure 3). The model-group mice’s colon was invaded by a multitude of inflammatory cells, leading to many necrotic lesions and crypt abscesses. Less inflammatory cell infiltration and crypt structural damage were seen in the lactulose (general drug dosage) group. Despite some mild inflammatory infiltration in the LP-HFY11 group, the crypt structure was mostly unaltered. When compared to low doses of LP-HFY11, high quantities of protein significantly decreased pathological damage to colon tissue. The LP-HFY11 treatment reduces the effect of constipation on the colon and is more effective than the pharmaceutical lactulose.

### 3.5. Mouse Serum Indicators

The normal group had considerably greater blood levels of MTL, ET-1, VIP, and AchE (*p* < 0.05) than the other groups (Table 4), with the model group exhibiting the lowest levels. The blood levels of MTL, ET-1, VIP, and AchE declined successively in the LP-HFY11H, lactulose, and LP-HFY11L groups. The blood levels of MTL, ET-1, VIP, and AchE were greater in the model group than in the normal group.

### 3.6. mRNA Expression of Related Genes in the Mouse Small Intestine

**Figure 4:**
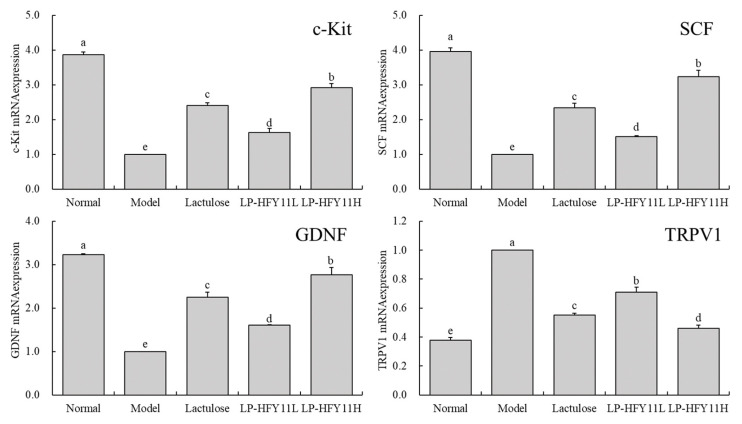
The c-Kit, SCF, GDNF, and TRPV1 mRNA expression of small intestine tissue in mouse (*n* = 10). ^a–e^ The data mean values between the groups show statistically significant differences (*p* < 0.05) indicated by the letters.

LP-HFY11 may substantially (*p* < 0.05) upregulate c-Kit, stem cell factor (SCF), and glial cell line-derived neurotrophic factor (GDNF) mRNA expression in the small intestine of the constipated mice relative to that of the animals in the model group, with the LP-HFY11H group exhibiting the highest degree of upregulation (Figure 4), compared with the model mice, the expression levels of c-Kit, SCF, and GDNF were 2.92, 3.23, and 2.76 times higher than model group, respectively. Additionally, transient receptor potential vanilloid 1 (TRPV1) expression was downregulated in the LP-HFY11 group than in the model group, bringing TRPV1 expression in the LP-HFY11H group closer to that in the normal group.

### 3.7. mRNA Expression in Mouse Intestinal Contents

**Figure 5:**
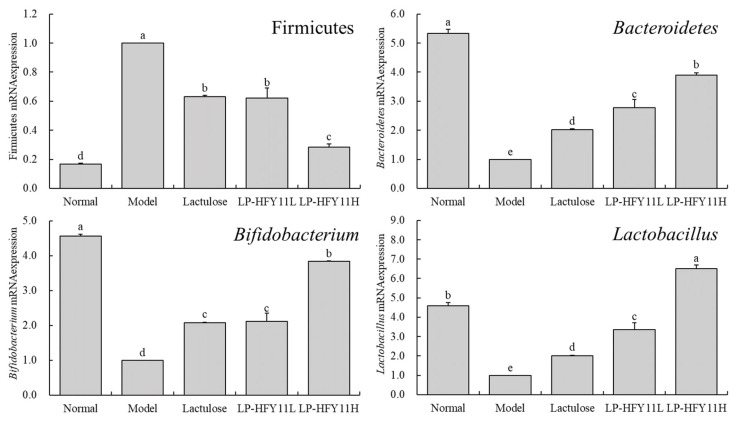
The *Firmicutes*, *Bacteroidetes*, *Bifidobacterium*, and *Lactobacillus* mRNA expression of intestinal contents in mouse (*n* = 10). ^a–e^ The data mean values between the groups show statistically significant differences (*p* < 0.05) indicated by the letters.

When analyzing the microbial mRNA expression intensity in the mouse gut contents, we noted that the proportion of *Firmicutes* bacteria was the lowest in the normal group (Figure 5). LP-HFY11 could reduce the proportion of *Firmicutes* bacteria among the intestinal microbes of the constipated mice. Meanwhile, the normal group exhibited the highest proportions of *Bacteroidetes* and *Bifidobacterium*. LP-HFY11H could make the proportions of *Bacteroidetes* and *Bifidobacterium* in the intestinal contents of the constipated mice closest to that in the normal group. Additionally, the intestinal contents of the LP-HFY11H group had the highest proportion of *Lactobacillus*.

## 4. Discussion

Constipated patients experience difficulty with defecation and have a lower frequency of bowel movements than healthy individuals. The most visible indicator of constipation severity is the state of feces. Fecal weight, fecal particle count, and fecal water content are all significant markers of fecal condition; a decline in these indices denotes a worsening of the degree of constipation [17]. According to research, giving rats and mice lactic acid bacteria after they have been constipated can help them reduce the quantity of fecal particles and water content that constipation causes and prevent constipation from having an adverse effect on their bodies [18]. According to this study, constipation mice’s fecal weight, particle number, and water content all increase when exposed to the LP-HFY11. By monitoring the amount of time taken to pass the activated charcoal water-created black stool in a constipated individual, the degree of constipation may be determined, as evidenced in the mouse constipation model. This constipation model mimics the condition in humans [19]. In this study, the time required by the constipated mice to excrete black stool after ingesting LP-HFY11, as well as the difficulty in defecating, were considerably reduced.

The interaction between probiotics and human gut microbiota is a dynamic and complex process, covering multiple levels of competition, metabolism, immune regulation, and intestinal barrier. Not only do they help maintain intestinal health, but they may also play an important role in the prevention and treatment of a variety of diseases. Therefore, moderate intake of probiotics is of great significance for improving human health [20]. The intestinal environment, immune system, enteric nervous system and central nervous system all participate in the regulation of intestinal motility. The enteric nervous system, central nervous system and immune system, intestinal secretion, intestinal flora and fermentation products are interconnected to regulate intestinal motility. Probiotics may help to regulate the enteric nervous system or the central nervous system to normalize intestinal motility and thus improve constipation. The gut microbiota also plays a crucial role in the normal development of the CNS, and they appear to interact with the CNS and gut via microbiota-enterochromaffin cell-vagal afferent nerve signals. At present, increasing evidence supports the existence of a bidirectional “microbial-gut–brain axis”, which plays a key role in regulating intestinal motility [21]. The use of probiotics to modulate microbiota–gut–brain interactions has been proposed as a novel approach for the treatment of intestinal motility disorders. A species of Lactobacillus reuteri has been shown to modulate neurally dependent motor reflexes that communicate with the brain in mice. Lactobacillus reuteri has also been shown to selectively increase the excitability of myenteric neurons in rats, suggesting that the mechanism of action of probiotics involves the enteric nervous system [22]. Furthermore, in an in vitro study, *Escherichia coli* Nissle fermentable supernatant could increase the maximal tone of human colonic smooth muscle, suggesting that *Escherichia coli* Nissle may affect intestinal contractility by directly stimulating smooth muscle cells [23]. The above studies have confirmed that specific microbial strains as probiotics can regulate and intervene in constipation by regulating nerve and intestinal function. The aim of this study is to find and verify strains that are beneficial to intestinal health and can intervene in constipation.

Activated charcoal may affect the normal movement of the intestine. Since activated carbon can adsorb water and nutrients in the intestine, it causes dry and reduced volume of intestinal feces, which may lead to slow intestinal peristalsis and cause constipation. Rapid discharge of activated charcoal is beneficial for intestinal health, and studies have shown that the rate of active discharge is closely related to intestinal health and constipation procedures. In mice, the degree of constipation may be established by measuring two noteworthy markers of the small intestinal function, namely the advancement distance and propulsion rate of activated charcoal in the small intestine. A longer advancement distance and a greater propulsion rate indicate reduced constipation [22]. This study clearly unveiled that the small intestine’s vitality changed significantly after the mouse constipation model was established. Moreover, substantial changes in the propulsion of activated charcoal were observed between the model and normal groups (*p* < 0.05). LP-HFY11 could significantly increase the propulsion rate of activated carbon in the small intestine, confirming that LP-HFY11 might also facilitate intestinal peristalsis and expel activated carbon that is prone to constipation, and this effect was related to the intake of LP-HFY11. Previous studies have shown that the rate of activated carbon expulsion is related to the degree of constipation [24], LP-HFY11 also can effectively relieve constipation by taking advantage of this function.

MTL, a gastrointestinal hormone, can encourage intestinal peristalsis and boost pepsinogen release, thereby aiding regular intestinal physiological processes and defecation [25]. ET-1 usually exists in the body at very low physiological concentrations and maintains the normal function of the body by regulating multiple organs. In the pathological process accompanied by vascular endothelial injury, a large amount of ET-1 is released, which causes damage to the central nervous system and neurons, constricts blood vessels and affects intestinal peristalsis. In severe cases, it may also cause intestinal obstruction [26]. A decline in VIP release might cause constipation because it relaxes smooth muscles and dilates blood vessels, thereby increasing the production of intestinal secretions and stimulating intestinal peristalsis [27]. AchE regulates intestinal contractions, which also facilitates mucus production. Augmenting mucus secretion and intestinal contractions aids in easier waste evacuation, thus averting constipation [28]. Constipation and intestinal flora disorder interact, and constipation leads to intestinal flora disorder, which in turn affects intestinal function and intestinal motility. At present, the clinical use of laxatives, gastric motility drugs and secretion drugs for the treatment of patients with constipation, although the short-term effect is good, but the long-term use is easy to affect the spontaneous recovery of defecation function of patients, and easy to lead to drug dependence, serious can induce electrolyte disorders and colon melanin. Therefore, it is necessary to find more reasonable, efficient and safe drugs for the treatment of patients with constipation. MTL, ET-1, VIP, and AchE are enteric neurotransmitters and gastrointestinal hormones. Now some specific drugs can inhibit constipation by interfering with these enteric neurotransmitters and gastrointestinal hormones [29]. At the same time, some animal experiments have also verified that probiotics can inhibit constipation by regulating MTL, ET-1, VIP, and AchE [30,31]. The present study also confirmed that serum levels of MTL, ET-1, VIP, and AchE may decrease in mice as the degree of constipation increases. LP-HFY11 was highly successful in treating constipation, as evidenced by the considerable increase in MTL, ET-1, VIP, and AchE levels induced by LP-HFY11 in this experiment compared with the model group. Furthermore, the magnitude of increase with LP-HFY11 was larger than that observed with lactulose.

The interstitial cells of Cajal (ICC) are crucial mediators of the communication between smooth muscles and the enteric nervous system. They can control the neurological signaling of smooth muscle cells. According to clinical research results, ICC levels in the body drop during constipation, and because the c-kit is a particular ICC marker, using it as a criterion for monitoring ICC levels is crucial [32]. ICC can only survive at certain concentrations of the SCF. They cannot be cultured or survive at a very high SCF concentration; hence, high SCF expression levels are detrimental for ICC survival [33]. TRPV1 is closely related to absorption and defecation. When activated, TRPV1 can trigger neurotransmitter release, which thus leads to motility disorders of the small intestine. At the same time, increased TRPV1 expression is a critical indicator of intestinal injury. Because intestinal damage triggers intestinal motility disorder, TRPV1 expression in the small intestine is more pronounced during constipation [34]. In addition to protecting and repairing injured nerve fibers, GDNF regulates neural cell growth and development, which is advantageous for repairing the damaged intestine and avoiding constipation [35]. Clinical studies and animal experiments have confirmed that constipation leads to the reduction in ICC in the colon of patients or experimental animals. Regulating the expression of c-kit and SCF can increase ICC, thereby relieving constipation [36,37]. At the same time, in vivo experiments have also verified that probiotics can regulate the expression of intestinal TRPV1, kit, SCF, and GDNF, and play a role in inhibiting constipation and restoring intestinal health [18]. In this study. LP-HFY11 also downregulated TRPV1 expression and efficiently upregulated the expression of c-kit, SCF, GDNF, and other factors, which assisted in avoiding and controlling constipation. The results of this study are consistent with previous clinical and animal studies, suggesting that specific probiotics can regulate the expression of gut related genes to inhibit constipation.

Intestinal microbiota dysbiosis is a critical factor leading to constipation. The human gut is mainly composed of *Firmicutes* and *Bacteroidetes*. When a larger number of *Firmicutes* than *Bacteroidetes* causes an imbalance in the intestinal microecology, blood levels of inflammatory substances and the risk of intestinal tissue lesions might increase [38]. *Bifidobacteria* aid in regulating intestinal immunity, maintaining normal physiological functions of the gut by affecting immune cells and cellular factors, and strengthening the barrier function of the intestinal mucosa, which supports in preventing constipation-related symptoms [39]. Studies have revealed that *Lactobacilli* can reduce the pH of the digestive tract, thus promoting the growth of beneficial bacterial flora and suppressing the growth of harmful bacteria. By augmenting the balance of the intestinal microecology, *Lactobacillus* can promote intestinal health and alleviate constipation [40,41]. Furthermore, the metabolic products of this genera can stimulate the motility of intestinal smooth muscles, increase gastrointestinal motility, and facilitate fecal movement through the intestine. *Lactobacillus* can also aid in increasing the fecal water content, softening stool, and thereby relieving constipation symptoms [42]. Thus, LP-HFY11 had a good regulatory effect on the intestinal microbiota, which resulted in healthier intestinal microecology in the constipated mice and alleviated constipation-induced intestinal dysbiosis. A previous study showed that LP-HFY11 has a strong anti-acid and bile salt effect in vitro [11], suggesting that it can effectively colonize the intestine and exert probiotic effects. The reason why lactic acid bacteria have strong resistance in vitro is that the special proteins and polysaccharides in the cell membrane can resist the acidic environment, and the special lipids and proteins in the cell membrane can enhance the resistance to bile salts [43]. The excellent resistance of LP-HFY11 in vitro may also be due to the composition of the cell membrane, and further studies on the cell composition of LP-HFY11 will become an important direction of its mechanism of action.

## 5. Conclusions

To examine the intervention impact of LP-HFY11 on constipation, a mouse model of diphenoxylate-induced constipation. The experimental results revealed that LP-HFY11 can alleviate constipation-induced defecation difficulties, physiological indicators, and intestinal flora dysbiosis. LP-HFY11, a lactic acid bacterium isolated from food, functions better than the drug lactulose (general drug dosage), which is frequently used for constipation treatment, and exerts no negative effects. LP-HFY11 can also regulate the intestinal flora to relieve constipation, aligning more appropriately with the physiological metabolism of the body, and therefore has better application prospects. However, this study first verified the intervention effect of LP-HFY11 on constipation through animal experiments, but there is still a lack of in-depth analysis on the mechanism of LP-HFY11. In the future, it is necessary to conduct in-depth research on the mechanism of LP-HFY11 and explore the relationship between Lp-HFy11’s own genes and the effect of inhibiting constipation. In addition, this study is limited to animal experiments, and clinical human experiments are needed to verify the effect in the future, and to provide better support for the food and biomedical industries.

## Figures and Tables

**Table 1:** Primer sequence for the reverse transcription–polymerase chain reaction.

Gene	Primer Sequence
TRPV1	F: 5′-CCGGCTTTTTGGGAAGGGT-3′
	R: 5′-GAGACAGGTAGGTCCATCCAC-3′
GDNF	F: 5′-TCAGCCATCACAGTGTTCCC-3′
	R: 5′-ATAGCCCGCATAGCGTATCAG-3′
c-Kit	F: 5′-AGACCGAACGCAACTT-3′
	R: 5′-GGTGCCATCCACTTCA-3′
SCF	F: 5′-AAACTGGTGGCGAATC-3′
	R: 5′-CACGGGTAGCAAGAAC-3′
	R: 5′-GTGCTCCGGTTGTATAAGATGAC-3′
GAPDH	F: 5′-AATGGATTTGGACGCATTGGT-3′
	R: 5′-TTTGCACTGGTACGTGTTGAT-3′
*Firmicutes*	F: 5′-GCGTGAGTGAAGAAGT-3′
	R: 5′-CTACGCTCCCTTTACAC-3′
*Bacteroidetes*	F: 5′-ACGCTAGCTACAGGCTTAACA-3′
	R: 5′-ACGCTACTTGGCTGGTTCA-3′
*Lactobacillus*	F: 5′-CACCGCTACACATGGAG-3′
	R: 5′-AGCAGTAGGGAATCTTCCA-3′
*Bifidobacterium*	F: 5′-TCGCGTCYGGTGTGAAAG-3′
	R: 5′-CCACATCCAGCRTCCAC-3′
Total bacteria	F: 5′-ACTCCTACGGGAGGCAGCAGT-3′
	R: 5′-ATTACCGCGGCTGCTGGC-3′

**Table 2:** Fecal weight, particle number, and water content of mice during the experiment (*n* = 10).

Group	Normal	Model	Lactulose	LP-HFY11L	LP-HFY11H	LP-YS3L	LP-YS3H	LP-CQPC10L	LP-CQPC10H
1–6 day (diphenoxylate suspension treated, samples untreated)									
Fecal weight (g)	0.90 ± 0.06 ^a^	0.48 ± 0.06 ^b^	0.50 ± 0.07 ^b^	0.47 ± 0.05 ^b^	0.46 ± 0.06 ^b^	0.46 ± 0.03 ^b^	0.47 ± 0.05 ^b^	0.48 ± 0.04 ^b^	0.47 ± 0.07 ^b^
Fecal particle number	36 ± 3 ^a^	21 ± 4 ^b^	23 ± 5 ^b^	21 ± 5 ^b^	23 ± 2 ^b^	22 ± 2 ^b^	23 ± 4 ^b^	23 ± 3 ^b^	22 ± 3 ^b^
Fecal water content (%)	49.7 ± 3.5 ^a^	14.7 ± 3.8 ^b^	14.9 ± 4.1 ^b^	14.6 ± 4.4 ^b^	14.9 ± 4.3 ^b^	14.6 ± 2.9 ^b^	14.8 ± 3.2 ^b^	14.7 ± 4.1 ^b^	14.8 ± 3.9 ^b^
7–12 day (samples treated)									
Fecal weight (g)	0.95 ± 0.06 ^a^	0.46 ± 0.07 ^d^	0.75 ± 0.06 ^b^	0.71 ± 0.06 ^b^	0.86 ± 0.05 ^ab^	0.59 ± 0.05 ^c^	0.72 ± 0.05 ^b^	0.70 ± 0.04 ^b^	0.85 ± 0.07 ^ab^
Fecal particle number	39 ± 4 ^a^	20 ± 4 ^c^	30 ± 3 ^a^	29 ± 4 ^ab^	35 ± 4 ^a^	24 ± 3 ^bc^	28 ± 2 ^b^	28 ± 3 ^b^	33 ± 3 ^a^
Fecal water content (%)	50.1 ± 3.6 ^a^	14.4 ± 3.1 ^d^	34.6 ± 4.5 ^c^	32.7 ± 3.7 ^c^	42.6 ± 3.9 ^b^	27.6 ± 3.8 ^c^	33.1 ± 4.0 ^c^	31.9 ± 3.3 ^c^	40.5 ± 3.9 ^b^

^a–d^ The data mean values between the groups show statistically significant differences (*p* < 0.05) indicated by the letters.

**Table 3:** Promotion of activated charcoal in mouse small intestine (*n* = 5).

Group	Small Intestine Length (cm)	Propulsion Distance (cm)	Propulsion Rate (%)
Normal	46.5 ± 3.7 ^a^	46.5 ± 3.7 ^a^	100.00 ± 0.00 ^a^
Model	44.9 ± 4.1 ^a^	10.8 ± 2.9 ^e^	24.99 ± 3.70 ^e^
Lactulose	45.6 ± 3.3 ^a^	28.7 ± 3.5 ^c^	64.31 ± 11.36 ^c^
LP-HFY11L	45.1 ± 3.8 ^a^	19.5 ± 3.1 ^d^	45.07 ± 8.47 ^d^
LP-HFY11H	45.9 ± 3.5 ^a^	35.1 ± 2.6 ^b^	75.83 ± 4.69 ^b^

^a–e^ The data mean values between the groups show statistically significant differences (*p* < 0.05) indicated by the letters.

**Table 4:** The serum MTL, ET-1, VIP, and AchE levels in mouse (*n* = 10).

Group	MTL (pg/mL)	ET-1 (pg/mL)	VIP (pg/mL)	AchE (nmol/L)
Normal	60.44 ± 4.32 ^a^	10.81 ± 1.57 ^e^	16.23 ± 1.44 ^e^	19.68 ± 1.67 ^a^
Model	23.25 ± 3.04 ^e^	28.12 ± 2.18 ^a^	40.15 ± 2.78 ^a^	2.42 ± 0.41 ^e^
Lactulose	46.56 ± 4.83 ^c^	17.72 ± 1.66 ^c^	27.21 ± 2.24 ^c^	12.04 ± 1.54 ^c^
LP-HFY11L	36.72 ± 3.81 ^d^	22.56 ± 1.79 ^b^	33.84 ± 2.22 ^b^	6.17 ± 1.36 ^d^
LP-HFY11H	53.18 ± 2.98 ^b^	15.25 ± 1.57 ^d^	23.63 ± 2.04 ^d^	15.59 ± 1.40 ^b^

^a–e^ The data mean values between the groups show statistically significant differences (*p* < 0.05) indicated by the letters.

## Data Availability

The data presented in this study are available on request from the corresponding author.
